# Moderating Effects of Alexithymia on Associations between the Therapeutic Alliance and the Outcome of Brief Psychodynamic-Interpersonal Psychotherapy for Multisomatoform Disorder

**DOI:** 10.3389/fpsyt.2017.00261

**Published:** 2017-12-04

**Authors:** Thomas Probst, Heribert Sattel, Harald Gündel, Peter Henningsen, Johannes Kruse, Gudrun Schneider, Claas Lahmann

**Affiliations:** ^1^Georg-Elias-Müller Institute for Psychology, Georg-August University of Göttingen, Göttingen, Germany; ^2^Department for Psychotherapy and Biopsychosocial Health, Danube University Krems, Krems, Austria; ^3^Department of Psychosomatic Medicine and Psychotherapy, Klinikum rechts der Isar, Technical University of Munich, Munich, Germany; ^4^Department of Psychosomatic Medicine and Psychotherapy, Ulm University, Ulm, Germany; ^5^Department of Psychosomatic Medicine and Psychotherapy, University of Giessen, Giessen, Germany; ^6^Department of Psychosomatic Medicine and Psychotherapy, University of Marburg, Marburg, Germany; ^7^Department of Psychosomatics and Psychotherapy, University Hospital Münster, Münster, Germany; ^8^Department of Psychosomatic Medicine and Psychotherapy, University Medical Center Freiburg, Freiburg, Germany

**Keywords:** alexithymia, therapeutic alliance, psychodynamic psychotherapy, somatic symptom disorder, outcome

## Abstract

This secondary analysis of a trial on brief psychodynamic-interpersonal therapy (PIT) for patients with multisomatoform disorder investigated whether alexithymia moderates the associations between the therapeutic alliance and the outcome of PIT and whether moderating effects of alexithymia remain significant when controlling for depression. Eighty-three patients with multisomatoform disorder receiving PIT were statistically analyzed. Moderation analyses were performed with the SPSS macro PROCESS. The primary outcome (Y), self-reported physical quality of life at 9-month after the end of PIT, was measured with the physical component summary (PCS) of the SF-36 Health Survey. The potential moderator (M) alexithymia was operationalized with the Toronto Alexithymia Scale (TAS-20) at pre-treatment and the predictor (X) the therapeutic alliance was rated by both patients and therapists *via* the Helping Alliance Questionnaire (HAQ) at the end of PIT. Moreover, the PCS at pre-treatment functioned as covariate in all moderation models. When the patients’ alliance ratings were analyzed, alexithymia did not moderate associations between the alliance and the outcome. When the therapists’ alliance ratings were evaluated, alexithymia moderated the relationship between the alliance and the outcome (*p* < 0.05): a stronger alliance in the therapists’ perspective was beneficial for the outcome only for patients scoring above 61 on the TAS-20. This moderating effect of alexithymia was, however, not statistically significant anymore when adding the pre-treatment depression scores (PHQ-9) as a covariate to the moderation model. The results underline the importance of a good therapists’ view of the alliance when treating alexithymic patients and highlight the complex interaction between alexithymia and depression. Future studies are needed to extend the scope of research regarding which psychotherapeutic mechanisms of change are beneficial for which patients.

## Introduction

Brief psychotherapies are efficacious for patients with multiple medically unexplained physical symptoms [e.g., Ref. (1)]. However, some patients with functional somatic syndromes undergoing psychotherapy also experience negative effects that can be attributed to factors within and outside the context of the psychotherapy (2). Patient variables have been shown to contribute to a more positive or more negative psychotherapy. For example, the short-term outcome of cognitive behavioral therapy (CBT) was more positive for patients suffering from somatoform disorders when they had a psychiatric history, higher psychological symptom severity, less characteristics related to personality-disorder, and a higher mental quality of life (3). Leibbrand et al. (4), however, failed to show associations between the treatment outcome of patients with somatoform disorder and their comorbid anxiety, depression, and personality disorders. In another study, long-term treatment outcomes were more negative for patients with somatization syndrome when the patients had a low acceptance of psychotherapy and less treatment expectations (5). Another patient variable associated with a less favorable outcome of psychodynamic psychotherapy is alexithymia (6). In CBT, alexithymia either did not influence the outcome (7) or had a beneficial effect on the outcome (8). Alexithymia can be described as having difficulties in identifying and describing emotions as well as by an externally oriented thinking style and affect regulation deficits (9–11). Karukivi and Saarijärvi (12) reviewed factors related to the development of alexithymia and identified genetic, environmental, and individual developmental factors. Some studies have shown a correlation between alexithymia and symptoms related to somatization [e.g., Ref. (11, 13)]. Although this association remained significant even when controlling for depression in the study by Mattila et al. (13), other studies reported that this correlation diminishes when controlling for negative affect such as depression [e.g., Ref. (14–16)].

In psychotherapy, therapists show predominately contempt when working with alexithymic patients (17). Such reactions of the therapists might contribute to the detrimental effect alexithymia exerts on the outcome of some psychotherapies (18). Moreover, alexithymia correlated negatively with the therapeutic alliance, which in turn correlated positively with the outcome in a current study on CBT and interpersonal therapy for patients with depressive disorder (8). A strong therapeutic alliance is associated with a more favorable psychotherapy outcome across all patients (19); yet, some patients benefit more than others from a strong therapeutic alliance. For example, Lorenzo-Luaces et al. (20) found that the therapeutic alliance affected the outcome only in depressed patients with 0–2 prior episodes but not in depressed patients with at least three prior episodes. Zilcha-Mano and Errázuriz (21) reported that symptom severity moderated the alliance-outcome link with more severely distressed patients benefiting more from a strong alliance.

To investigate alexithymia as a moderator of the alliance-outcome relationship, the current study re-analyzed data from a multicenter randomized controlled trial (RCT) on brief psychodynamic-interpersonal therapy (PIT) for patients with multisomatoform disorder (22). A multisomatoform disorder diagnosis requires at least three current, functionally disabling somatoform symptoms (on at least half of the days over at least 2 years) not sufficiently explained by an organic disease or another mental disorder, and intensive health-care use (23). Although this research question was not initially planned in the context of the RCT, we hypothesized that the therapeutic alliance exerts a more beneficial effect on the outcome of PIT in patients with higher alexithymia than in patients with lower alexithymia. This hypothesis bases on findings that therapists experience more difficulties in the relationship with alexithymic patients (8, 17, 18) suggesting that it is more important to reduce these difficulties/to establish a strong alliance in alexithymic than non-alexithymic patients. Moreover, we investigated whether alexithymia moderates the relationship between the alliance and the outcome of PIT even when controlling for depression. We had no specific hypothesis here because research on the question whether alexithymia and depression overlap has produced inconsistent results [e.g., Ref. (24, 25)].

## Materials and Methods

The multicenter randomized controlled trial [“PISO trial” (22)] the data was drawn from was registered (ISRCTN23215121) and approved by the ethics committees of the six participating sites. Informed consent was obtained from all patients.

### Study Procedure

In the PISO trial, *n* = 107 patients were randomized to the intervention condition (PIT) and *n* = 104 patients were allocated to the control condition (enhanced medical care, EMC). A blocked randomization list (stratified random blocks of four, six, or eight patients) was generated and applied to the sample by the Coordination Centre for Clinical Trials. The follow-up assessment was realized 9-month after the end of the treatment by post.

### Patients

The patients met the diagnostic criteria for multisomatoform disorder (23) and suffered from pain as the predominant symptom. The “Structured Clinical Interview” (SCID) for DSM-IV (26) was used and modified according to the criteria published by Kroenke et al. (23) to diagnose a multisomatoform disorder. Retrospectively, all patients fulfilled the criteria for a somatic symptom disorder according to DSM-5.

### Treatment

The patients of the present study received manualized brief psychodynamic-interpersonal psychotherapy [PIT (27)]. The control condition of the PISO trial (EMC) was not analyzed in the study at hand, since forming a therapeutic alliance was of particular importance in the intervention as compared to the control condition: establishing a therapeutic alliance was an explicit component of the PIT protocol but not of the EMC protocol [see study protocol published as supplement in Ref. (28)].

PIT consisted of 12 weekly individual sessions including the establishment of the therapeutic alliance, the treatment of the somatoform symptoms, their behavioral, emotional, and interpersonal correlates, and the discussion of termination issues (22, 27, 29). Delivery of PIT was controlled for adherence by independent raters (30). Moreover, therapists used checklists to rate their adherence to the PIT manual for each session.

### Measures

The primary outcome was patient-reported physical quality of life at 9-month after the end of PIT (22) and it was operationalized with the Physical Component Summary (PCS) of the SF-36 Health Survey (31). Moreover, patients filled in the Toronto Alexithymia Scale [TAS-20 (9)] at pre-treatment to measure alexithymia. Furthermore, the therapeutic alliance was rated by patients and therapists with the Helping Alliance Questionnaire [HAQ (32)] at the end of PIT; patients but not therapists filled in the HAQ also at 9-month after PIT; therefore, the ratings at the end of PIT were statistically analyzed to have patients’ and therapists’ alliance ratings measured at the same assessment point. Furthermore, the patients’ scores on the depression scale of the Patient Health Questionnaire [PHQ-9 (33)] at pre-treatment were used in the present study to investigate whether the potential moderating effect of alexithymia on the alliance-outcome link is robust even when controlling for depression.

### Statistics

To explore whether alexithymia moderates associations between the patients’/therapists’ alliance ratings and the outcome, and whether this is also the case after controlling for depression, moderation models were performed with PROCESS (34). PROCESS is a SPSS macro for moderation and mediation analysis. Within PROCESS, model 1 was selected and the confidence interval was set to 95%. In the moderation models, the alliance ratings (HAQ) at the end of PIT were entered as the predictor (X), physical quality of life (PCS) at 9-month after PIT functioned as the outcome (Y), alexithymia at pre-treatment (TAS-20) was added as the moderator (M), and physical quality of life (PCS) at pre-treatment was entered as covariate (to analyze the PCS change from pre-treatment to 9-month after PIT). In further models, depression (PHQ-9) at pre-treatment was added as second covariate. In case a statistically significant interaction between the predictor the therapeutic alliance (X) and the moderator alexithymia (M) emerged, the Johnson–Neyman Technique was applied to identify the threshold(s) of the moderator (M) where the association between the predictor (X) and the outcome (Y) transition(s) between statistical significance and non-significance (34). All statistical tests were performed two-tailed and the significance value was set to *p* < 0.05.

## Results

### Sample Description

**Figure 1:**
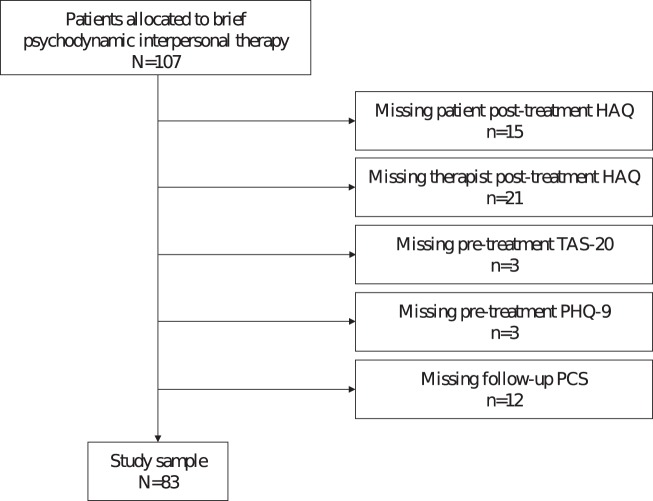
Flow-chart. Note: More missing questionnaires than excluded patients because of multiple missing questionnaires per excluded patient. Abbreviations: HAQ, Helping Alliance Questionnaire; TAS-20, Toronto Alexithymia Scale; PHQ-9, Depression scale of the Patient Health Questionnaire; PCS, Physical Component Summary of the SF-36 Health Survey.

Patients’ and therapists’ HAQ ratings, patients’ TAS-20 scores, patients’ PHQ-9 scores, as well as patients’ PCS scores were available for *N* = 83 of all *N* = 107 patients receiving PIT. The flow-chart is presented in Figure 1.

These 83 patients (gender: 61.4% female; age: M = 48.34, SD = 11.27) were treated per protocol by eight therapists and represent the sample of the current study.

### Correlations between Specific Measures

The therapists’ ratings of their adherence to PIT did not significantly correlate with the TAS-20 scores for any of the 12 sessions (*r* ranged from −0.18 to 0.11; all *p* > 0.12). The correlations between the TAS-20 and the HAQ ratings did not reach statistical significance either (patients’ HAQ: *r* = −0.08; *p* = 0.46; therapists’ HAQ: *r* = −0.07; *p* = 0.53). Yet, the TAS-20 scores were significantly positively related to the PHQ-9 scores (*r* = 0.41; *p* < 0.01). The difference scores of the PCS (pre-treatment scores were subtracted from follow-up scores) did not significantly correlate with either the TAS-20 scores (*r* = −0.18; *p* = 0.10) or the HAQ ratings (patients’ HAQ: *r* = −0.16; *p* = 0.15; therapists’ HAQ: *r* = 0.06; *p* = 0.58) across all patients.

### Alexithymia as a Moderator of the Alliance-Outcome Link without Taking Depression into Account

#### Patients’ Alliance

Table 1 shows that the interaction effect between the patients’ alliance ratings and patients’ alexithymia on physical quality of life at 9-month after PIT did not attain statistical significance (*t* = 0.58; *p* = 0.57). Therefore, alexithymia did not moderate the association between the patients’ alliance ratings and the outcome.

**Table 1:** Results of the moderation analysis investigating alexithymia as a moderator of the association between the patients’ alliance ratings and the outcome.

Outcome: PCS at follow-up						
**Model summary**						
***R***	***R*^2^**	**MSE**	***F***	**df1**	**df2**	***p*-Value**

0.57	0.32	66.40	9.27	4.00	78.00	<0.01

	**Model**					
	**Coefficient**	**SE**	***t***	***p*-Value**	**LLCI**	**ULCI**

Constant	32.16	18.35	1.75	0.08	−4.38	68.69
TAS-20	−0.35	0.39	−0.90	0.37	−1.12	0.42
HAQ_P	−3.60	3.90	−0.92	0.36	−11.36	4.17
HAQ_P*TAS-20	0.05	0.08	0.58	0.57	−0.12	0.21
PCS at pre-treatment	0.88	0.15	6.01	<0.01	0.59	1.17

**Conditional effect of HAQ_P on PCS at follow-up at values of TAS-20**						
**TAS-20 values**	**Effect**	**SE**	***t***	***p*-Value**	**LLCI**	**ULCI**

10th percentile: 31	−2.11	1.55	−1.36	0.18	−5.19	0.98
25th percentile: 40	−1.67	1.08	−1.55	0.13	−3.83	0.48
50th percentile: 49	−1.24	1.03	−1.20	0.23	−3.30	0.82
75th percentile: 56	−0.91	1.33	−0.68	0.50	−3.55	1.74
90th percentile: 62	−0.62	1.70	−0.36	0.72	−4.01	2.77

*PCS, Physical Component Summary of the SF-36 Health Survey; TAS-20, Toronto Alexithymia Scale; HAQ_P, Helping Alliance Questionnaire patient version; LLCI, lower level of the confidence interval; ULCI, upper level of the confidence interval*.

#### Therapists’ Alliance

The interaction effect between the therapists’ alliance ratings and patients’ alexithymia on physical quality of life at 9-month after PIT reached statistical significance (*t* = 2.01; *p* < 0.05; see Table 2). This means that alexithymia moderated the association between the therapists’ alliance ratings and the outcome. Reported in the lower part of Table 2, a stronger therapists’ alliance exerted a significantly beneficial effect on the outcome only at very high values of alexithymia (for the 90th percentile of the TAS-20 scores: *t* = 2.01; *p* < 0.05). The moderator value defining the Johnson–Neyman significance region was a TAS-20 score of 61.21 (% below: 85.54; % above 14.46).

**Table 2:** Results of the moderation analysis investigating alexithymia as a moderator of the association between the therapists’ alliance ratings and the outcome.

Outcome: PCS at follow-up						
**Model summary**						
***R***	***R*^2^**	**MSE**	***F***	**df1**	**df2**	***p*-Value**

0.58	0.34	64.75	10.01	4.00	78.00	<0.01

	**Model**					
	**Coefficient**	**SE**	***t***	***p*-Vaue**	**LLCI**	**ULCI**

Constant	52.66	20.86	2.52	0.01	11.13	94.20
TAS-20	−0.98	0.44	−2.24	0.03	−1.86	−0.11
HAQ_T	−8.68	4.85	−1.79	0.08	−18.33	0.98
HAQ_T*TAS-20	0.21	0.10	2.01	<0.05	0.002	0.41
PCS at pre-treatment	0.83	0.14	5.79	<0.01	0.55	1.12

**Conditional effect of HAQ_T on PCS at follow-up at values of TAS-20**						
**TAS-20 values**	**Effect**	**SE**	***t***	***p*-Value**	**LLCI**	**ULCI**

10th percentile: 31	−2.26	1.93	−1.17	0.25	−6.10	1.58
25th percentile: 40	−0.40	1.34	−0.30	0.77	−3.06	2.27
50th percentile: 49	1.47	1.25	1.17	0.25	−1.03	3.97
75th percentile: 56	2.92	1.61	1.81	0.07	−0.29	6.13
90th percentile: 62	4.16	2.07	2.01	<0.05	0.03	8.29

*PCS, Physical Component Summary of the SF-36 Health Survey; TAS-20, Toronto Alexithymia Scale; HAQ_T, Helping Alliance Questionnaire therapist version; LLCI, Lower level of the confidence interval; ULCI, Upper level of the confidence interval*.

### Alexithymia as a Moderator of the Alliance-Outcome Link When Controlling for Depression

#### Patients’ Alliance

As summarized in Table 3, alexithymia did also not moderate the association between the patients’ alliance at the end of PIT and physical quality of life at 9-month after PIT when adding depression at pre-treatment as a covariate to the moderation model (*t* = 0.38; *p* = 0.71).

**Table 3:** Results of the moderation analysis investigating alexithymia as a moderator of the association between the patients’ alliance ratings and the outcome when controlling for depression.

Outcome: PCS at follow-up						
**Model summary**						
***R***	***R*^2^**	**MSE**	***F***	**df1**	**df2**	***p*-Value**

0.57	0.33	66.56	7.57	5.00	77.00	<0.01

	**Model**					
	**Coefficient**	**SE**	***t***	***p*-Value**	**LLCI**	**ULCI**

Constant	30.37	18.48	1.64	0.10	−6.42	67.17
TAS-20	−0.24	0.40	−0.60	0.55	−1.05	0.56
HAQ_P	−2.92	3.98	−0.74	0.46	−10.84	4.99
HAQ_P*TAS-20	0.03	0.09	0.38	0.71	−0.14	0.20
PCS at pre-treatment	0.85	0.15	5.69	<0.01	0.55	1.15
PHQ-9 at pre-treatment	−0.17	0.18	−0.91	0.37	−0.53	0.20

**Conditional effect of HAQ_P on PCS at follow-up at values of TAS-20**						
**TAS-20 values**	**Effect**	**SE**	***t***	***p*-Value**	**LLCI**	**ULCI**

10th percentile: 31	−1.92	1.56	−1.23	0.22	−5.04	1.19
25th percentile: 40	−1.63	1.08	−1.50	0.14	−3.79	0.53
50th percentile: 49	−1.34	1.04	−1.29	0.20	−3.41	0.73
75th percentile: 56	−1.12	1.35	−0.83	0.41	−3.80	1.57
90th percentile: 62	−0.92	1.74	−0.53	0.60	−4.38	2.54

*PCS, Physical Component Summary of the SF-36 Health Survey; TAS-20, Toronto Alexithymia Scale; HAQ_P, Helping Alliance Questionnaire patient version; PHQ-9, Depression scale of the Patient Health Questionnaire; LLCI, lower level of the confidence interval; ULCI, upper level of the confidence interval*.

#### Therapists’ Alliance

Alexithymia did not significantly moderate the association between therapists’ alliance at the end of PIT and physical quality of life at 9-month after PIT anymore when depression at pre-treatment was added as a covariate to the moderation model (see Table 4; *t* = 1.83; *p* = 0.07).

**Table 4:** Results of the moderation analysis investigating alexithymia as a moderator of the association between the therapists’ alliance ratings and the outcome when controlling for depression.

Outcome: PCS at follow-up						
**Model summary**						
***R***	***R*^2^**	**MSE**	***F***	**df1**	**df2**	***p*-Value**

0.58	0.34	65.45	7.95	5.00	77.00	<0.01

	**Model**					
	**Coefficient**	**SE**	***t***	***p*-Value**	**LLCI**	**ULCI**

Constant	51.28	21.25	2.41	0.02	8.97	93.59
TAS-20	−0.92	0.47	−1.98	0.05	−1.85	0.01
HAQ_T	−8.21	5.01	−1.64	0.11	−18.18	1.76
HAQ_T*TAS-20	0.20	0.11	1.83	0.07	−0.02	0.41
PCS at pre-treatment	0.82	0.15	5.55	<0.01	0.53	1.11
PHQ-9 at pre-treatment	−0.08	0.18	−0.41	0.68	−0.44	0.29

**Conditional effect of HAQ_T on PCS at follow-up at values of TAS-20**						
**TAS-20 values**	**Effect**	**SE**	***t***	***p*-Value**	**LLCI**	**ULCI**

10th percentile: 31	−2.13	1.96	−1.09	0.28	−6.04	1.77
25th percentile: 40	−0.37	1.35	−0.28	0.78	−3.05	2.31
50th percentile: 49	1.39	1.27	1.09	0.28	−1.14	3.93
75th percentile: 56	2.77	1.66	1.66	0.10	−0.54	6.07
90th percentile: 62	3.94	2.15	1.83	0.07	−0.34	8.23

*PCS, Physical Component Summary of the SF-36 Health Survey; TAS-20, Toronto Alexithymia Scale; HAQ_P, Helping Alliance Questionnaire patient version; PHQ-9, Depression scale of the Patient Health Questionnaire; LLCI, lower level of the confidence interval; ULCI, upper level of the confidence interval*.

## Discussion

This study re-analyzed data from a multicenter randomized controlled trial on PIT for multisomatoform disorder to investigate whether alexithymia moderates associations between the therapeutic alliance and the outcome of PIT. Across all patients, neither alexithymia [in contrast to Ref. (6)] nor the therapeutic alliance [contrary to Ref. (19)] correlated with the outcome of PIT. However, alexithymia moderated the effect a stronger therapists’ alliance exerted on the outcome of PIT: significantly beneficial effects of a stronger therapists’ alliance on the outcome emerged only for patients scoring above the TAS-20 threshold of 61 points (9, 35) diagnostic for clinically relevant alexithymia. These results fit to other studies highlighting the role of the person of the therapist when working with alexithymic patients (6, 17, 18). However, alexithymia was no significant moderator of the relationship between the therapists’ alliance and the outcome anymore when controlling for depression. The non-significant effect of alexithymia when taking depression into account is in line with other studies reporting that symptoms related to somatization did not correlate anymore with alexithymia [e.g., Ref. (14, 16)] or associated affect regulation deficits (15) when controlling for negative affect such as depression. Yet, this was not the case in the study by Mattila et al. (13). It has been discussed that alexithymia and depression are overlapping constructs [e.g., Ref. (24, 25)] and the correlation between alexithymia and depression became also significant in the current study (*r* = 0.41) fitting to the moderate relationship (*r* = 0.43) found in the meta-analysis by Li et al. (36). Another study investigating alexithymia as a moderator between psychotherapeutic mechanisms of change and the outcome did not control for depression but for general symptom distress: Terock et al. (37) found that the patients’ psychotherapy motivation (more specifically “degree of suffering”) improved the short-term outcome only for patients with lower TAS-20 scores (37). As a stronger alliance in the therapists’ view was beneficial for patients with higher TAS-20 scores in the present study (before controlling for depression), it could be speculated that different psychotherapeutic mechanisms of change have a different impact on the outcome depending on the patients’ level of alexithymia. However, such conclusions have to be drawn with caution, since the sample used to analyze interactions between motivation, alexithymia, and the outcome was more heterogeneous and received a more multimodal/less manualized (psychiatric day hospital) treatment (37) than the outpatient sample of the present study on interactions between the alliance, alexithymia, and the outcome. More research is needed to inform therapists which psychotherapeutic mechanism/s of change [alliance, motivation, resource activation, problem actuation, mastery, clarification, insight, installation of hope, expectations, therapy techniques, …; see for example, Ref. (38–41)] is/are more or less beneficial for which kind of patients within a diagnostic category.

The result that alexithymia did not moderate—before and after controlling for depression—associations between the patients’ alliance ratings and the outcome might be attributable (at least to a certain extent) to the lacking ability of patients with higher alexithymia to adequately rate the relationship with the therapist due to their emotion-processing deficits.

One limitation of the present study is that the therapeutic alliance was assessed only once at the end of the intervention. The therapeutic alliance can fluctuate during psychotherapy and it would be more appropriate to measure the alliance on a session-to-session basis. Another shortcoming of the present study is the solely use of a self-rating to assess alexithymia. Although the TAS-20 is a frequently used alexithymia questionnaire, observer-based ratings might be less biased measures of alexithymia (11). A further limitation is the correlational design with its associated threats to the internal validity. One potential confounder in the context of the present study might be a difference in the therapists’ treatment adherence between patients with higher and lower alexithymia, especially regarding the PIT component “establishment of a therapeutic alliance.” However, the non-significant correlations between the therapists’ adherence ratings and patients’ alexithymia for all 12 PIT sessions indicate that treatment adherence was independent from alexithymia. The results that the therapists’ as well as the patients’ ratings of the therapeutic alliance were not significantly associated with alexithymia—contrary to Quilty et al. (8)—offer further evidence that therapists did not differ in their adherence to the PIT component “establishment of a therapeutic relationship” between patients with higher and lower alexithymia. Nevertheless, there are numerous other potential confounders and the internal validity of the results would have been stronger if patients with higher and also patients with lower alexithymia had been randomized to either a PIT condition including the component “establishment of the therapeutic relationship” or to a PIT condition excluding this component. Yet, it is also questionable whether it is ethical and feasible to conduct such component studies with regard to the therapeutic alliance.

## Ethics Statement

The multicenter randomized controlled trial (“PISO trial”) the data was drawn from was registered (ISRCTN23215121) and approved by the ethics committees of the six participating sites. Informed consent was obtained from all patients.

## Author Contributions

TP drafted and revised the manuscript, contributed to the study design, and performed statistical analyses; HS revised the manuscript, contributed to the study design, and performed statistical analyses; HG revised the manuscript and contributed to the study design; PH was the grant recipient of the PISO trial, revised the manuscript, and contributed to the study design; JK revised the manuscript and contributed to the study design; GS revised the manuscript and contributed to the study design; CL drafted and revised the manuscript and contributed to the study design.

## Conflict of Interest Statement

The authors declare that the research was conducted in the absence of any commercial or financial relationships that could be construed as a potential conflict of interest.
